# Predator cue-induced plasticity of morphology and behavior in planthoppers facilitate the survival from predation

**DOI:** 10.1038/s41598-021-96005-3

**Published:** 2021-08-18

**Authors:** Jian Wen, Takatoshi Ueno

**Affiliations:** Institute of Biological Control, Faculty of Agriculture, Kyushu University, Fukuoka, 819- 0395 Japan

**Keywords:** Agroecology, Evolutionary ecology, Evolution

## Abstract

Predators can induce phenotypic plasticity in prey through selection driven by predation risk. However, defense plasticity is rarely reported in insects, let alone trans-generational plasticity, meaning the mechanisms underlying plasticity, how it impacts ecosystem evolution and how it might be exploited in pest control are poorly understood. Here we examine the morphological plasticity of small brown planthoppers (SBPHs), *Laodelphax striatellus*, elicited by caged predators, *Paederus fuscipes* in the parent or F1 generation and reveal the risk cues mediating these effects. We also uncover the survival outcomes in SBPHs with predator-induced defensive morphological traits by examining their survival probability and behavioral plasticity. Results showed that caged predators or predator odor cue gave rise to a higher proportion of long-winged, female SBPHs in the parent and F1 generations, but the proportion of males and their wing length were unaffected. The visual cue from predators elicited weaker effects. Surprisingly, we discovered these long-winged forms suffered a lower predation rate when attacked by *P. fuscipes,* owing to an enhanced agility level. Our results suggest the within- and trans-generational plasticity of induced defenses may cause profound effects on SBPH population dynamics and prey-predator interaction. Understanding this interaction and its underlying mechanisms illuminates important aspects of ecosystem evolution and helps predict pest dispersal or migration, which in turn may be exploited for pest control.

## Introduction

Insects adjust adaptively their morphological, behavioral or physiological traits in fast-changing environments^1,2^, a characteristic that has allowed insects to thrive on our planet for millions of years. Such adaptive traits have been well documented and include dispersal or migration^3,4^, hibernation or diapause^5^, oviposition site selection^6^, metabolic regulation^7^, and immune response^8^. Thus, insects can exhibit a wide range of environmental adaptation from immediate reactions to slower, but deeper, physiological changes. Furthermore, such adaptations are commonly linked to defense against predators. In many organisms, including insects, anti-predator defense has been a focus of attention in animal biology^9^. More recently, several organisms have been demonstrated to exhibit flexible anti-predator defenses in the form of morphological plasticity. Such morphological plasticity in defense emerges when organisms are under predation stress, i.e.*,* non-consumptive effects. Examples include smaller body mass in birds^10^, larger body mass in treefrogs^11^, changes in body shape in fish^12^, thicker shells in snails^13^, and better-developed defensive spikes in *Daphnia*^14^, demonstrating the importance of non-consumptive effects by predators on the morphological plasticity of prey organisms. However, similar phenomena have only been reported in some species of insects and so we have limited knowledge about flexible defense responses in the world’s most abundant group of organisms. Further, the mediation mechanisms of predation risk effects are still not clear, with the cues (odor or /and visual cue) that induce such morphological plasticity remaining largely undetermined.

Doubtless, flexible defense should be an important strategy enabling insects to escape from predation, and that the types of flexible response will likely vary greatly in time and space. Unlike predictable factors, *i.e.,* seasonal changes that can be sensed from photoperiods and temperatures, predation itself may happen unexpectedly or unpredictably. To cope with predation risks, insects have developed a set of flexible defenses. For example, non-myrmecophilous butterflies avoid laying eggs on plant branches with a higher number of ant visits because ants would predate their larvae^15^. Also, studies have shown insect pollinators avoid visiting flowers when artificial spiders are present on the flowers^16^. Surprisingly, the tobacco hornworm caterpillar reduces its leaf consumption when the predation risk from stink bugs rises, but its assimilation efficiency and development rate rise concurrently. This allows it to reduce the risk of predation while still maintaining growth^17^. With these flexible defenses, insects can increase their chance of survival and reproductive success under predation. Furthermore, flexible defense strategies may vary between sexes because female and male responses may differ depending on their vulnerability to predation. For example, male stalk-eyed flies have a greater eye span and display more aggression towards a predator than females because their poorer flight capability makes them easier prey^18^. However, such sexual differences in defense have not been reported widely in insects, given that sexual dimorphism evolves through mate choice and male-male fights are common in insect communities^19^.

Organisms may also exhibit transgenerational plasticity. This occurs when maternal effects are carried from one or more prior generation(s) to influence the phenotypes of offspring^20,21^. A growing body of evidence suggests transgenerational plasticity may be as common as within-generational plasticity, and may occur across all kinds of defensive traits^22^. Transgenerational plasticity of inducible defense has been well reported in non-insect groups^22^. With such induced transgenerational defense plasticity, threatened parents can increase the fitness of their offspring by transferring their risk experience to the next generation, or by influencing the phenotypes of their offspring to suit the predation environment they are likely to experience. Transgenerational plasticity in defense has seldom been examined in insects, however, demonstration of such plasticity would expand our understanding of how anti-predation defense, or non-consumptive strategies, express over time, and so may be important in the evolution in insects.

Both within- and trans-generational defense plasticity should be costly^22^, because organisms need an allocation of energy, or resource, to form defensive traits, which in turn reduces the energy or resources that can be used for other purposes, i.e., development or reproduction^23,24^. This conflict should have important implication for pest management, as predator-induced phenotypic plasticity alters the development, survival, reproduction, population dynamics, and interspecific interactions of pests, resulting in profound and broad consequences not only on pest communities, but also on the whole agroecosystems^25–27^.

The small brown planthopper (*Laodelphax striatellus*, hereafter SBPH) is a virus-carrying rice pest of economic importance^28,29^. SBPH has two forms^30,31^: brachypter (short-winged form) and macropter (long-winged form). Brachypters are less mobile and have a higher reproductive capacity, while macropters have a stronger flight ability that enables them to emigrate when food supplies deteriorate or population overcrowding occurs, but are less fecund. This is a typical example of what is known as wing polymorphism in insects. The rove beetle *Paederus fuscipes* is one of the most abundant generalist predators in rice paddies, and is distributed widely in Asia^32^. Under laboratory conditions, we have recorded that one female *P. fuscipes* is able to consume 12–15 3rd instar SBPH nymphs, or 6–10 4th or 5th instar nymphs, or 5–8 long-winged adults per day (unpublished data). Certainly, *P. fuscipe* signals that it presents a strong predation risk for SBPHs because it is an actively searching hunter with a brightly-colored body. Given the abundance and potential importance of this predatory beetle in rice paddies, SBPHs should have evolved a defensive strategy against it, such as developing pathways to detect the presence of *P. fuscipe* (via odor cues or/ and visual cues) and then responding defensively. Very few studies however have examined the defensive strategies of SBPHs (and other planthoppers) against such generalist predators.

In this study, we reveal the links between wing polymorphism of SBPHs and predation risks, and the cues mediating this relationship. We demonstrate that previously threatened SBPHs show a stronger defensive response than that exhibited by those that have no threat experience (non-threatened), a phenomenon that has seldom been examined previously. This paper focuses on four questions: (1) do predation risks posed by *P. fuscipes* affect the wing polymorphism of SBPHs in the parent and F1 generations? (2) What are the cues mediating the morphological plasticity of SBPHs? (3) Do threatened parents or offspring survive better (have they gained greater fitness) when attacked by *P. fuscipes* than those that have never experienced prior predation risk? (4) How are SBPHs able to increase their survivability when attacked? We highlight that predation risk serves as an indispensable factor affecting how SBPHs respond flexibly to predation risk, and discuss how the predation risk of *P. fuscipes* might be used to control SBPHs. Lastly, we discuss how predation risk effects presented in SBPH could be universal in many insect species, and so act as one of the major forces influencing prey–predator interaction and evolution of insect.

## Results

### Proportion of macropters, surviving female SBPHs after being threatened

**Figure 1:**
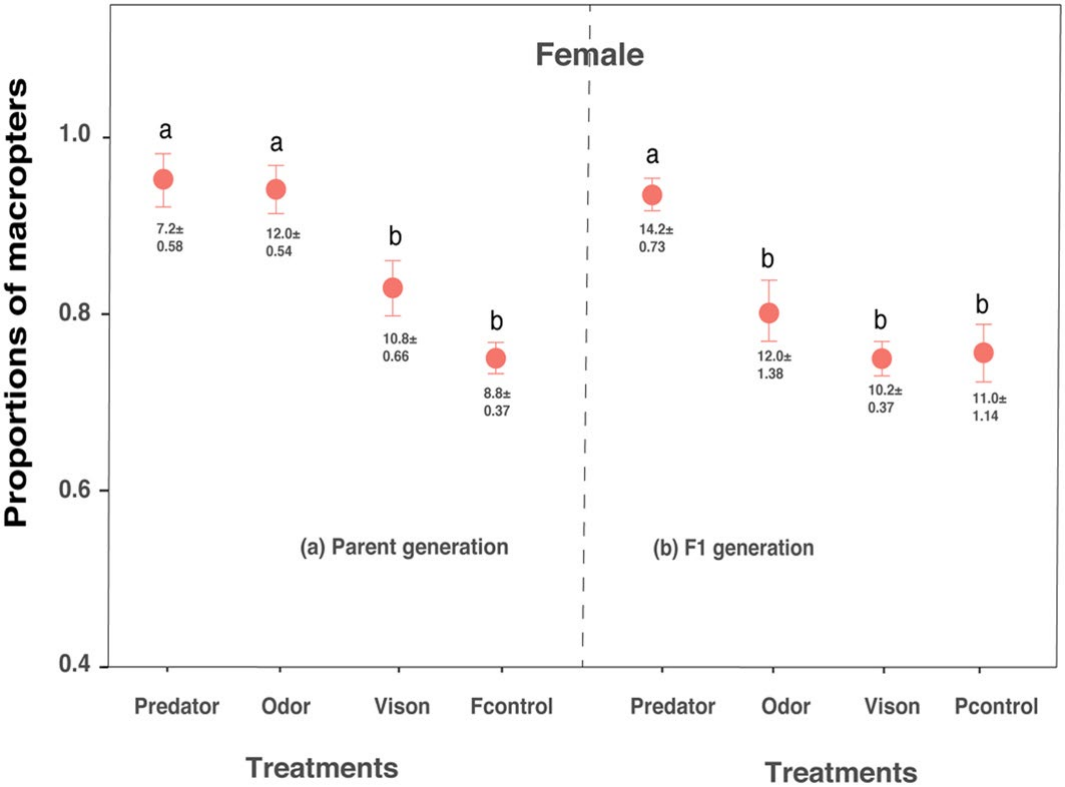
Effects of predation risks on the proportion (mean ± SE) of female macropters in the parent **(a)** and F1 **(b)** generations. The proportions of female macropters and brachypters are not independent of each other in that when the proportion of female macropters is higher, the other is lower. Predator: caged rove beetles; odor: odor cue from rove beetle body extract; vision: visual cue from sealed rove beetle cadavers. Pcontrol and Fcontrol indicate non-threatened macropters from parent and F1 controls respectively. Value annotations below the error bar indicate the mean surviving number of female macropters (mean ± SE); initial number of nymphs is forty. Different lowercase letters above the error bars denote significant differences among treatments (Tukey’s test, P < 0.05).

**Figure 2:**
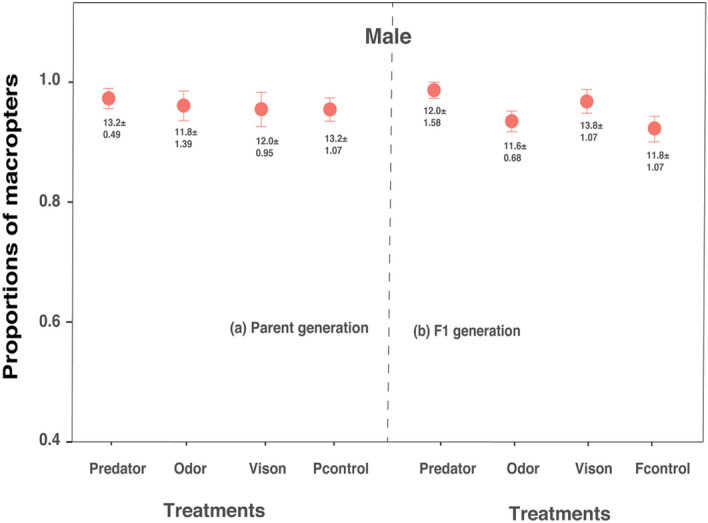
Effects of predation risks on the proportion (mean ± SE) of male macropters in the parent **(a)** and F1 **(b)** generations. The proportions of male macropters and brachypters are not independent of each other in that when the proportion of male macropters is higher, the other is lower. Predator: caged rove beetles; odor: odor cue from rove beetle body extract; vision: visual cue from sealed rove beetle cadavers. Pcontrol and Fcontrol indicate non-threatened macropters from parent and F1 controls respectively. Value annotations below the error bar indicate the mean surviving number of male macropters (mean ± SE); initial number of nymphs is forty. Different lowercase letters above the error bars denote significant differences among treatments (Tukey’s test, P < 0.05).

**Figure 3:**
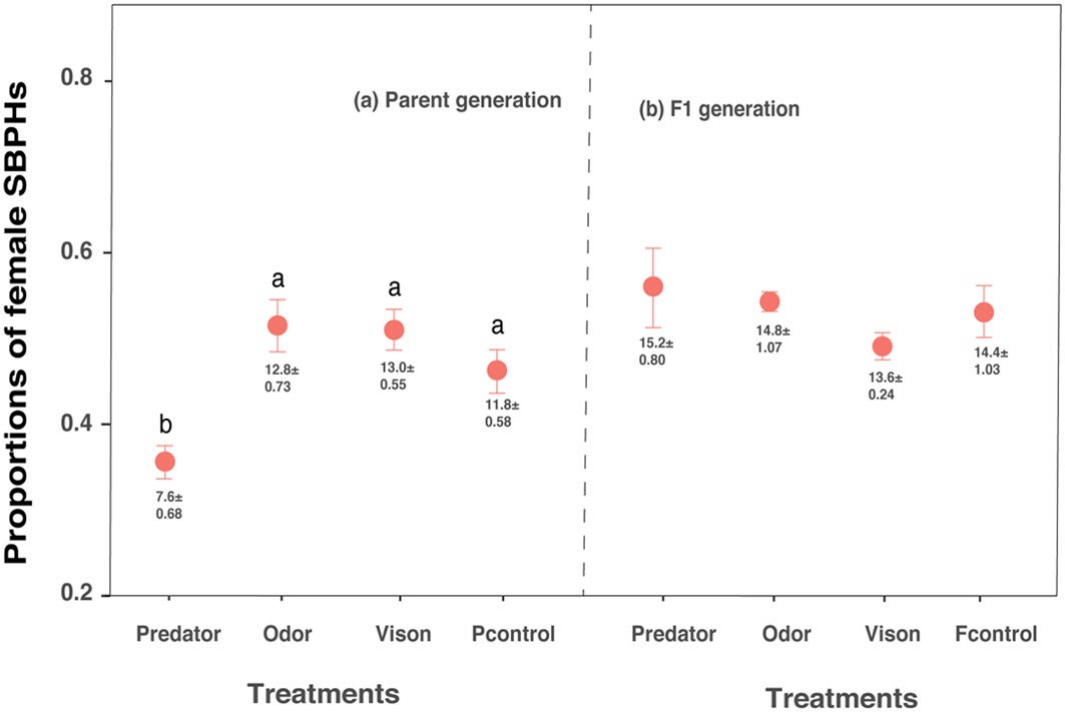
Effects of predation risks on the proportion (mean ± SE) of surviving female SBPHs in the parent **(a)** and F1 **(b)** generations. The proportions of females and males are not independent of each other in that when the proportion of females is higher, the other is lower. Predator: caged rove beetles; odor: odor cue from rove beetle body extract; vision: visual cue from sealed rove beetle cadavers. Pcontrol and Fcontrol indicate non-threatened macropters from parent and F1 controls respectively. Value annotations below the error bar indicate the mean number of female macropters (mean ± SE); initial number of nymphs is forty. Different lowercase letters above the error bars denote significant differences among treatments (Tukey’s test, P < 0.05).

The proportions of macropters when they were exposed to different predation risks (risk cues) is shown in Figs. 1 and 2. In the parent generation, a significantly higher proportion of nymphs treated with caged predators and predator odor cues developed into female macropters, but the proportion of nymphs treated with sealed cadavers (visual cue) was not significantly different from the control (Table 1a; Fig. 1a). In the F1 generation, only caged predators affected the proportion of nymphs developing into macropters (Table 1b; Fig. 1b). The proportion of male macropters in all treatments was no different from that in the control both in parent and F1 generations (Fig. 2a,b). The proportion of surviving female SBPHs was also affected by predation risks (parent generation: F_3,16_ = 8.38, p = 0.0014; F1 generation: F_3,16_ = 1.08, p = 0.385). In the parent generation, when nymphs were exposed to caged predators, a lower proportion of females developed (Fig. 3a). However, in the F1 generation, the proportion of surviving females was no different from those of the control (Fig. 3b).

**Table 1:** Results of two-way ANOVA of the proportion of macropters when exposed to different predation risks in the parent (a) and F1 (b) generations. Risk cues indicate exposure to caged rove beetles, odor cues from the rove beetle body extract, visual cues from the sealed rove beetle cadavers and control. Bold values indicate significant p-values (p < 0.05).

Variances	(a) Parent generation			(b) F1 generation		
	Df	F-value	P	Df	F-value	P
Risk cues	3	4.426	**0.010**	3	7.908	** < 0.001**
Sexes	1	13.99	** < 0.001**	1	49.24	** < 0.001**
Risk cues × sexes	3	2.611	0.068	3	1.677	0.192

### Survivorship of macropters when attacked by *P. fuscipes*

**Figure 4:**
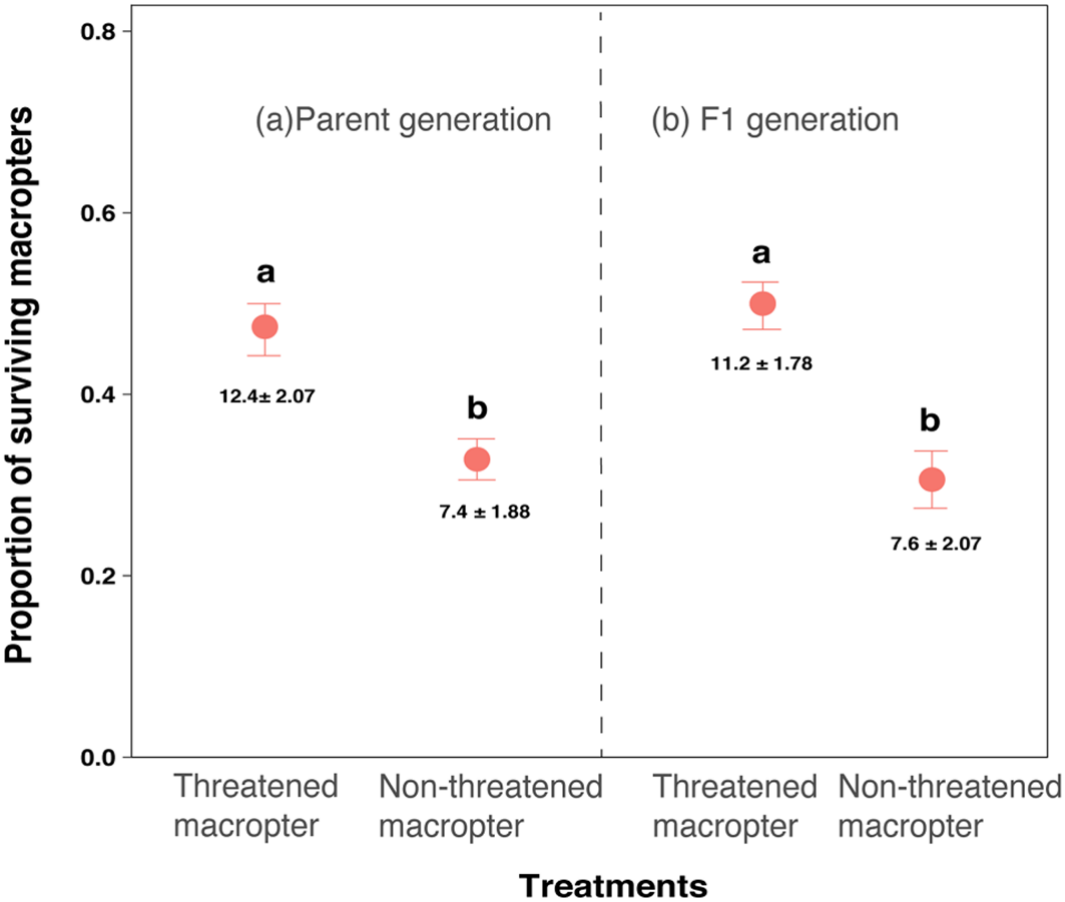
Effects of predation risks on the proportion (mean ± SE) of surviving macropters when attacked by *P. fuscipes* in the parent **(a)** and F1 **(b)** generations. Threatened macropter indicates macropters exposed to caged rove beetles, and non-threatened macropter indicates control macropters without risk exposure. The values inside the bar indicate the surviving number of macropters (mean ± SE). Different lowercase letters above the bars denote significant differences (Tukey’s test, P < 0.05) among treatments.

Prior exposure to predation risk influenced the survivorship of SBPHs when attacked by *P. fuscipes* (F_3,16_ = 16, P < 0.001, Fig. 4)*.* Already threatened macropterous parents and offspring had significantly higher survival rates than those that had not been exposed previously to predation risk (Fig. 4). More than 47% of previously threatened macropterous parents survived, though this proportion was not significantly different from that of their offspring (≈ 50%), but for both of them survivorship was significantly higher than for previously non-threatened macropters in parent (≈ 33%) and F1 generation (≈ 30%) controls.

### The agility level and survival analysis of macropters when attacked by *P. fuscipes*

**Figure 5:**
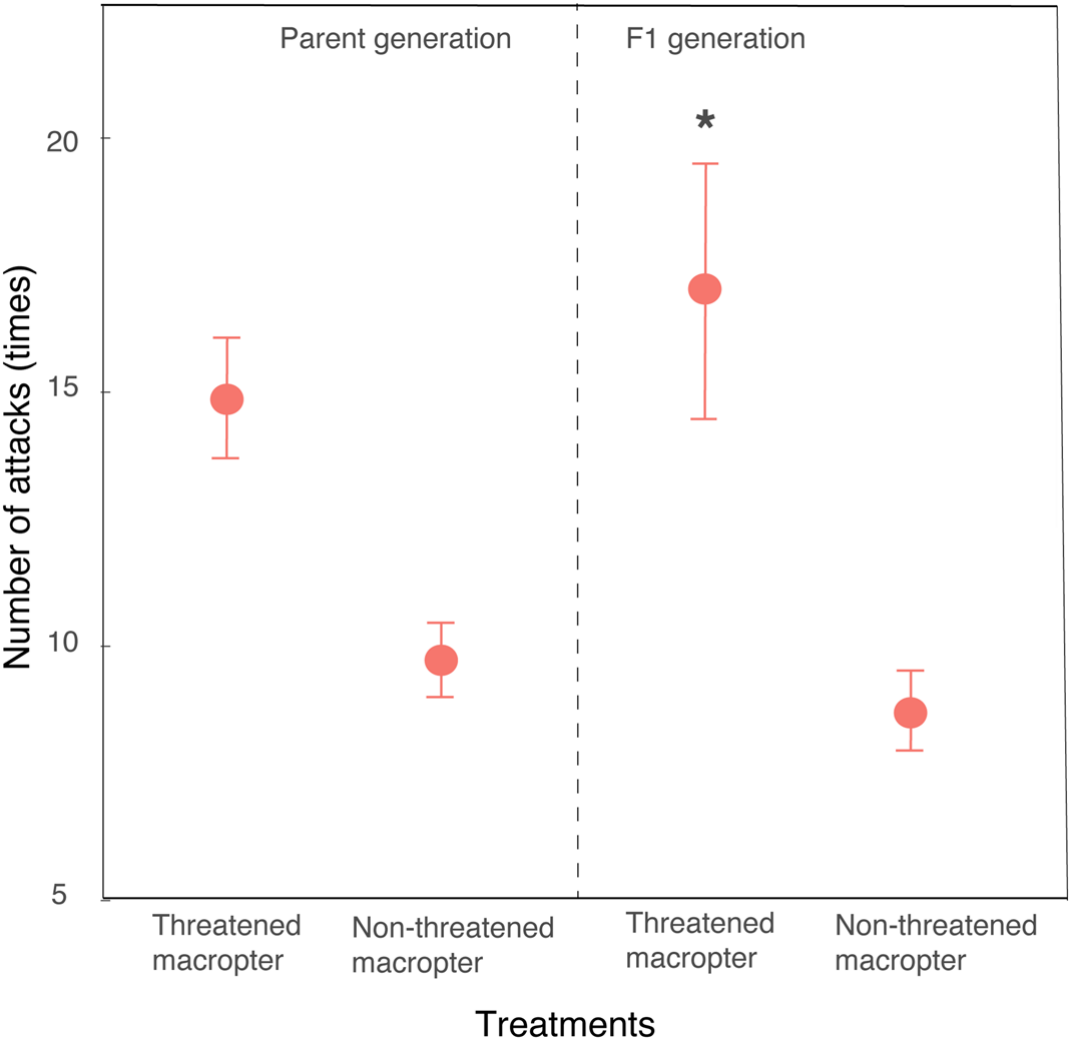
The number of attacks for *P. fuscipes* to capture one previously threatened or non-threatened macropter from the parent and F1 generations. Threatened macropter indicates macropters exposed to caged rove beetles, and non-threatened macropter indicates control macropters without risk exposure. The single asterisk above the error bar denotes a significant difference from the control (Wilcoxon test, P < 0.05).

**Figure 6:**
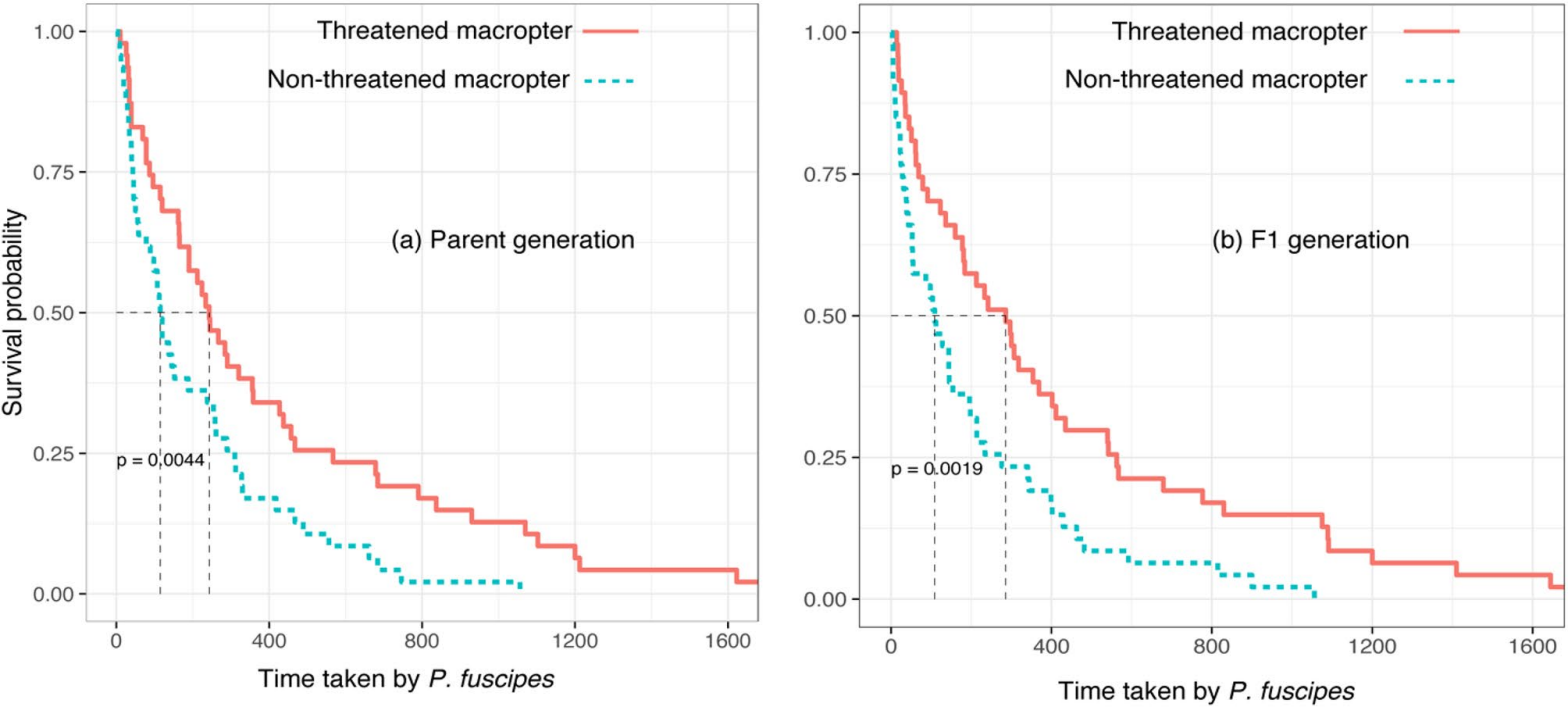
Survival analysis of the previously threatened and non-threatened macropters in the parent **(a)** and F1 **(b)** generations when attacked by *P. fuscipes.* Threatened macropter indicates macropters exposed to caged rove beetles, and non-threatened macropter indicates control macropters without risk exposure. The significance of differences between threatened and non-threatened macropters was measured using a log-rank test (P < 0.05).

The agility level and survival probability of macropters were affected by prior predation risk, but not by their sex (see Supplementary Table S2). *P. fuscipes* needed a significantly higher number of attacks to capture previously threatened macropterous offspring than for capturing non-threatened ones (Fig. 5), but this difference was not significant in the parent generation and between sexes (see Supplementary Table S2). For survival analysis, in the parent generation, the time at which 50% of previously threatened macropters were captured was 243 s, significantly higher than the time for macropters that had not been previously threatened (115 s; Fig. 6a; log-rank test: p = 0.0044). However, survival probability of macropters was no different between sexes (see Supplementary Table S3). The situation was similar in the F1 generation. Fifty percent of previously threatened macropters were captured within 286 s, however, it was only 109 s for macropters that had experienced no prior threat. Also, survival probability of pre-threatened macropters in the F1 generation was significantly higher than non-threatened macropters (Fig. 6b; log-rank test: p = 0.0019).

## Discussion

To defend against predators, insects often modify their morphology, flexibly, to enhance survival and reproductive advantages. Here, we report that predation risks from either isolated predator or predator odor cues, induce a higher proportion of nymphs to developed into long-winged females among the parent generation, as well as among F1 generation offspring. Surprisingly, these previously threatened long-winged adults survived better when attacked by a predator owing to the enhanced agility level gained from risk experience. The long wing, and increased agility level, provide adaptive benefits for SBPHs to escape from predation and so are able to go on to reproduce.

SBPHs responded more strongly to the caged predators (visual + odor risk cues) and predator odor cues, than just the visual cue of the predator. Different risk cues can elicit different levels of responses in prey^33–36^. For example, in the case of the Colorado potato beetle, volatile odor cues from the predator stronger reduced the beetle feeding on plants than predator visual and tactile cues^35^. But a visual cue has been shown to be crucial for insect pollinators detecting and avoiding flowers with predators^37^. Insect herbivores frequently communicate via chemical odors^33,38^. Exploiting the odor cue to perceive the presence of predators should have advantages, because the odor cue can be sensed from a long distance and penetrate the blocking effect of foliage or canopy structure^39^, enabling the prior detection of risks and the preparation of antipredation behaviors.

In densely planted rice paddies, the active foraging behavior of rove beetle may serve as a selective pressure favouring the development in SBPHs of a chemical instead of visual pathway to detect the approach of a rove beetle. However, in the F1 generation, the influence of a predator odor cue on the proportion of winged forms was weaker than that of caged predators, indicating the combined effects of odor and visual cues might be stronger than only an odor cue, suggesting that visual cues cannot be ignored. In our experiments, sealed predator cadavers may have weakened the visual cue of the rove beetles, because the lack of motion did not fully represent the normal visual cue.

SBPHs frequently exhibit wing plasticity in response to population density and food quality^28,29^. When nymph density is higher, or food has deteriorated, a higher proportion of macropters will arise^28,29^. The development of the winged form is thought to be a strategy for SBPHs to emigrate from inhospitable environments. However, we assumed, predation risks could also induce the occurrence of the winged form, because long wings might enable SBPHs to escape from predation. As expected, the results presented here show that a higher proportion of long-winged females and their offspring arose when nymphs or adults were previously exposed to predation risk, demonstrating that SBPHs can express morphologically plastic defenses in response to prior predation risk. Additionally, the higher proportion of wing forms was not only due to the increasing number of winged females (see Fig. 1, the number of winged females in “caged rove beetle” treatment was lower), but also the increasing proportion of winged females among female groups (the decreasing numbers and proportions of wingless females, Fig. 1). To date, similar patterns have only been shown in pea aphids, in which when predation risk (foot prints from lady beetles) is higher during the parent generation, a higher proportion of winged morphs arise in the offspring^40,41^. In our experiments, we tested the risk effects passing from nymphs to adults and from parents to their offspring with combined risk cues, an odor cue or a visual cue, which better reveals the capacity for flexible defense strategies within SBPHs and the nature of predation risks in the perpetual 'arms race' against predation. This is the first example of how insects can express both within- generational and transgenerational morphological plasticity as a defense strategy in response to prior predator threat, and we suggest that this phenomenon is likely to occur more widely.

However, SBPHs do not only face a single lethal pressure from their environment as we discussed above. Nymph density, food quality, even the temperatures or photoperiods may play or interplay roles in the induction of wing plasticity in SBPHs^28,29^. In these situations, the responses of SBPHs may differ from present results, or opposite results can occur. As an example, the growth rates of snails vary depending on snail densities, food supply and the strength of predation risks. Growth rates were higher when snails were reared on high nutrients and in low densities, but decreased steeply as the predation risk increased. Conversely, the growth rate was lower at high densities and with high predation risk, but increased as nutrient availability increased^42^. As for SBPHs, the proportion of winged adults may be higher if we reared in higher densities combined with high predation risk, or may be lower if the nutrient condition of the rice plants increases (for example, higher fertilizer inputs benefit the development of planthoppers^43^) and predators are removed. This hypothesis needs to be tested. Further, the rice plant phenotypes (resistant or sensitive phenotypes) are important to the development of planthoppers or leafhoppers^44–46^, and tests of the interactive effects of plant phenotype, plant quality/quantity, nymph density and predation risk on the wing plasticity of SBPHs should provide insights into the evolution of insects within changing environments.

Induced transgenerational defense plasticity as shown in SBPHs may be common in many organisms^20,47^. It allows parents to transfer their risk experience to offspring and promotes their evolutionary fitness^20^. When SBPH nymphs are exposed to predation risk, they are likely to develop into long-winged females, because it is an advantageous form for them in the current risk environment. However, such predation risk is variable in time and space, and SBPH parents cannot predict when or whether the predators will disappear. Thus, an appropriate strategy to enhance the survival rate of offspring in an unpredictable environment is to continue producing a higher proportion of long-winged forms. Within-generational and transgenerational plasticity of defense should be a successful adaptive defense strategy for SBPHs, given that rove beetle and other groups of predators such as predatory spiders are abundant all around the year in rice paddies.

The higher mortality of SBPH nymphs when they experience predation risk, has been broadly addressed before^24,48,49^. Reduced food intake during risk periods may contribute to this poorer survival outcome, because insects are likely to alter their feeding behavior^50,51^, or shift from a high-risk host to a safer, but nutritionally inferior, one^52^, when they detect the presence of predators. However, we did not observe an apparent behavior change in threatened nymphs in our experiments, even those going on to be macropters, compared to the non-threatened ones. For example, changing feeding location, non-feeding related motility, an increase in jump frequency, etc. did not occur in threatened nymphs. Thus, behavior plasticity seems not to be invoked to explain this phenomenon. However, considering the food consumption of sap-sucking SBPHs is difficult to determine, experiments employing electrical penetration graph (EPG) techniques should be conducted to quantify the amounts of sap consumption during risk periods^53^. This will help to explain whether the higher mortality is due to a change of feeding behavior (less food intake). Furthermore, some obscure internal physiological plasticity may also cause the higher mortality of SBPH nymphs at risk. For example, increased oxidative damage and decreased assimilation efficiency during the risk period may weaken the survival success of SBPH nymphs. Unfortunately, few studies have verified this assumption, although it has been shown that different assimilation efficiencies may arise under predation risk^17^, or oxidative damage may be induced by predation risk resulting in a slower growth rate^54^ and decreased escape performance^55^.

SBPHs exhibit sexual differences in both with- and trans- generational morphological plasticity in relation to defense, i.e.*,* threatened nymphs/parents are more likely to develop into long-winged females, due to the different vulnerability of females and males to predation. This predation difference is particularly acute between short-winged females and males, given that the proportion of short-winged females is lower than that seen in control settings (Fig. 1), and we assume the level of vulnerability may depend on their body size and reproductive role. The body sizes of short-winged females are larger than those of long-winged females or males, causing them to be more vulnerable to predation because they are more highly preferred targets for predator. Also, the short-winged female needs to stay and deposit eggs in the bare rice stem, which increases the time window of exposure to predators while, by contrast, long-winged males are slim and are not required to lay eggs, and so should be not be heavily predated. It follows that short-winged females should be more vulnerable to predation than long-winged females or males. Hence, in SHPBs, increasing the proportion of long-wing females in a population creates greater opportunities to migrate to predator-free habitats for reproduction, while at the same time reducing their vulnerability to predation. We hypothesize that the sexual difference in responses should be adaptive, and might be inheritable if predation pressure frequently favors the long-winged forms among populations over multiple generations.

Results presented here also show that previously threatened long-winged offspring survived better than previosuly non-threatened ones when attacked by *P. fuscipes*. Studies suggest prey-altered morphology in response to predation risks should confer a survival advantage (fitness gained), i.e., a better-developed defensive structure^13,24^, or refuge in having a larger size that increase survival success^57^. However, wings themselves are without protective functions for SBPHs, as seen in pea aphids^41^. Thus, we setup behavioral experiments to reveal how threatened long-winged adults may increase their survival when attacked by a predator. Results show threatened long- winged offspring (but not parents) are more active, and respond more quickly, than unthreatened ones, i.e., a higher number of attacks are needed for *P. fuscipes* to capture a previously threatened long-winged offspring than one that has not been threatened before. We suggest the increased agility level is not because of the long wing itself, but due to the enhanced muscle strength in the legs of long-winged adults, because in our observation, long-winged adults avoid attack mainly by jumping but not by flight, probably because a jump needs less reaction time than flight.

We only observed transgenerational plasticity of induced behavioral defense in SBPHs. This generational difference (within- and trans-generational) in behavioral defense in SBPHs may reflect potential carry-over effects from parents. To our knowledge, the generational difference in defense has rarely been shown in insects, though in pea aphids a fluctuating expression of transgenerational defensive traits (long wing) over generations when predation risk was present or absent has been reported^58^. We also expect there will be cumulative effects^59^ accumulated by SBPHs from the parent generation to the F1 generation. However, we are not certain whether these effects exist in our experiments. To determine this, experiments examining defensive traits across multigeneration should be conducted.

However, if predation risk increases the number of agile, long-winged SBPH adults, which are of benefit in respect of dispersal, migration, and thus spreading rice viruses, the application of *P. fuscipes* in biological control appears ultimately to weaken the control effectiveness. Also, a study with field experiments found that predatory ladybugs increase the number of dispersed aphid nymphs, especially in plants with lower resistance. However, surprising results show that the higher number of dispersed aphid nymphs will not necessarily translate into population growth because dispersed aphids are weak (less food intake) and more easily predated by predators^60^. Thus, the benefits of anti-predator defense in aphids will, over time, translate into negative developmental costs that suppress the aphid population. As for SBPHs, threatened long-winged females perform well in dispersal and defense, but worse in development and reproduction. Recent experiments reveal that previously threatened long-winged females have a longevity that is three days shorter, and produces about 60 fewer eggs per female, than non-threatened long-winged females (unpublished data). Consequently, these negative effects would eventually translate into lower population growth rates within SBPHs. Thus, the introduction of the predation risk from *P. fuscipes* to control SBPHs is workable, since field experiments in controlling western flower thrips and grasshoppers by exposure to predation risk have been successful^49,61^, and the main purpose of biological control is to suppress the pest population beneath the relevant economic threshold, and reduce plant mass loss without necessarily eliminating the pest altogether.

This study advances the importance of predation risk on the induction of flexible anti-predation defenses in insect parents and their offspring, uncovers the mediating mechanisms, shows how this anti-predation defense expresses differently between sexes, and further explores the adaptation significance of these defense traits for insects exposed to unpredictable environments. These findings should prove important for predicting SBPH migration or dispersal, conducting effective pest control, and better understanding prey-predator interactions. However, future work should examine the effects of predation risks from other groups of predators or parasites on the physiological and behavioral plasticity of SBPHs.

## Materials and methods

### Insects and equipment used to investigate predation risk effects

SBPHs were collected from wild weeds (mainly *Echinochloa crusgalli* (L.) P. Beauv) at Liji, West Ward, Fukuoka City, Japan in October 2018, and were reared in groups in a transparent square cage (30 × 25 × 35 cm, with meshed windows on the walls) together with 10- day potted rice seedlings (*Oryza* *sativa* L. ssp. *japonica*, 10–13 cm in height). The rice seeds we used here were provided by Plant Breeding Laboratory, Faculty of Agriculture, Graduate School, Kyushu University, at which this species has been traditionally used to rear planthoppers or leafhopper for a long time. Adult rove beetles (*Paederus fuscipes*) were collected from rice paddies at Kuwabara, West Ward, Fukuoka City, Japan, and were reared on mixed diets with collembolan insects, as well as SBPHs and brown planthoppers, in a transparent cylindrical tube (d = 12 cm, h = 20 cm, with meshed windows), again with rice plants. We used wild-caught rove beetles in these experiments because we could not maintain them over multiple generations in laboratory conditions. Rearing conditions for SBPHs and *P. fuscipes* both involved a photoperiod of 16 L:8 D, a temperature of 25 ℃, and 60% relative humidity, but they were kept separately in different incubators.

We produced predation risk effects of *P. fuscipes* on SBPHs with an experimental device (see Supplementary Fig. S1). This combined a transparent cylindrical tube (d = 12 cm, h = 20 cm, with meshed windows) and a round plastic box (d = 12 cm, h = 5 cm) connected beneath to the cylindrical tube. Rice seedlings and SBPHs were reared together in the device (see below). Experimental predator cues were put in a small meshed cage (d = 3 cm, mesh hole < 1 mm), and the cage containing predator cues was placed into the device. Thus, SBPHs could sense the odor and/or visual cue of *P. fuscipes*, but no direct contact or actual predation took place in the experimental set-up.

To separate the predation risks into a risk odor cue and a risk visual cue, predator (*P. fuscipes*) body extracts (odor cue) and sealed predator cadavers (visual cue) were prepared. The predator body extract was prepared by submerging one pair of frozen-killed rove beetles (1♀ + 1♂, starved for 24 h, at -10 °C for 10 min) in 15 mL distilled water for 24 h. This extract was stored at 4 °C for later tests. The sealed predator cadaver was prepared by carefully sealing a frozen- killed rove beetle (1♀ or 1♂, starved for 24 h, at − 10 °C for 10 min) with transparent cling film and waterproof glue. Airtightness detection of the sealed cadaver was checked by submerging the sealed cadaver in water for 60 s. The sealed cadaver that remained dry was deemed eligible and stored at 4 °C for later tests.

### Effects of predation risks on the proportion of macropters and survivorship in SBPHs

We hypothesized that predation risk during the nymph stage of SBPHs could cause them to develop into macropters, and mothers experiencing predation risk could enhance the proportion of macropters in the F1 generation, because the long-winged form should be advantageous for escaping from higher risk environments. To test our hypothesis, we setup experiments cross two generations. Thirty germinated rice seeds were sown in the 1 cm-depth of soil on the bottom of the device. Except for 20 rice seedlings that grew to a height of 10–13 cm (10 days) that were retained for use, the remaining seedlings were removed from the device.

For the parent generation, forty 1st instar nymphs were put into the device. A cage with one pair of 24 h starved *P. fuscipes,* or cotton wool soaked with 2 mL predator body extract, or two sealed predator cadavers (1♀ + 1♂) was then put into the device to produce odor and visual risk signals, or an odor signal or a visual signal, respectively. The initial density of 40 SBPHs (i.e.*,* less than 3 individuals per cm^2^) was selected in our experiment because this density was normally lower than in the field (3–5 individuals per cm^2^)^62^, which eliminated the possibility of disturbances from crowding effects on the test SBPHs. In control treatment (Pcontrol), 40 new-hatched 1st instar nymphs were reared in a device without risk cues (without *P. fuscipes,* or with wet cotton (distilled water), or with a piece of transparent film in the meshed cage).

For the F1 generation, in which the duration of risk exposure covered the parent nymph and adult stages, were also conducted. Our hypothesis was that a higher number of macropters would occur in the F1 generation when their parent had experienced a longer period of risk. In this experiment, SBPH nymphs were treated similarly to short-term risk exposures. After the tested nymphs had moulted to adults, 3–5 pairs of macropters were randomly selected for use, and were transferred to a new device with caged *P. fuscipes* or cotton soaked with 2 mL predator body extract or two sealed predator cadavers, to allow female macropters to lay eggs in the new rice seedlings. Forty 1st instar nymphs hatched from these eggs were collected immediately, and then reared in a new device without risk cues. These 1st instar nymphs were reared in this situation until emerging to the adult stage. In control treatment (Fcontrol), forty 1st instar nymphs were reared in the device without risk cues present for both the parent and the F1 generation.

In all the treatments, the cage, along with the *P. fuscipes,* or treated cotton, or sealed cadavers for both the parent generation and the F1 generation treatments were replaced every 2 days during the experiment. At the end of the experiments, we recorded the number of macropters and brachypters produced, together with their sex twice daily, and recorded adults would be removed from the device. All treatments were repeated 5 times.

### Survivorship of macropters when attacked by *P. fuscipes*

In this experiment, we tested whether the threatened macropters from parent and F1generations would survive better than those without risk experience. We set up experiments to compare the survival rate between threatened and non-threatened macropters when attacked by *P. fuscipes*. We did not test the survival of brachypters because they were produced rarely in our experimental conditions (see Supplementary Fig. S2). Also, we used 3rd instar nymphs in the initial stage because of the higher likelihood of 3rd instar nymphs developing to adults than 1st and 2nd instar nymphs when exposed to predation risk, and more of these adults were macropters, similar to using 1st and 2nd instar nymphs (see Supplementary Fig. S3). The threatened macropters from the parent generation, F1generation, and non-threatened macropters from controls (Pcontrol and Fcontrol) were produced as we described above, but we only used caged predators as the risk cues, because they represent the comprehensive risk effects on SBPHs. We waited until all nymphs had emerged, after which we collected all macropters and reared them in a device with one pair of non-caged, 24 h starved *P. fuscipes*. The survivorship of macropters in these four treatment groups was noted 72 h later. All treatments were repeated five times.

### The agility level and survivability of macropters when attacked by *P. fuscipes*

We explored what was the fitness gained by SBPH with an anti-predator trait (predator-induced long wing) and how risk experience could affect the ontogenic behavioral responses of macropters to the attack by *P. fuscipes*. The threatened macropters from the parent and F1 generation and non-threatened macropters from controls (Pcontrol and Fcontrol) we used here were produced by exposing 3rd instar nymph to caged predators (see above). Threatened or non-threatened macropters for testing were individually put into a transparent petri dish (d = 6 cm, h = 1.5 cm). We allowed them to calm for 5 min. One 12 h starved female *P. fuscipes* was then put into this petri dish, and the observation began. The observation was made over 30 min, or was terminated when the predator successfully captured the test SBPHs during the observation. The total number of attacks by, and the time taken, for, *P. fuscipes* to capture the macropters were examined. We set 50 replicates for each treatment (25 replicates of female macropters and 25 replicates of male macropters).

### Data analysis

All data analyses were conducted with R (version 4.0.3)^63^. We used Shapiro–Wilk tests to check the normality of data, while Bartlett tests were used to analyze the homogeneity of variances. As there are only two forms of SBPH, macropter and brachypter, their proportions are not independent of each other. For example, if more nymphs develop into macropter morphs, then fewer must develop into brachypter forms. The same applies when we calculate the proportions of surviving female and male SBPHs. Therefore, we only calculated the proportion of macropters and the proportion of surviving female SBPHs. The methods we used to calculate these proportion are showed in Supplementary Information (see Supplementary Methods and formulas). These proportions were arcsine transformed to meet the requirements of two/one- way ANOVA, followed by Tukey's test for multiple comparison. We also arcsine transformed the surviving proportion of macropters attacked by *P. fuscipes* and ran a one-way ANOVA (Tukey's test for multiple comparison). The number of attacks were analyzed by using generalized linear models (GLM) assuming a Poisson distribution, and a log link function to model the effects of predation risk, sex, and their interactions, on the number of attacks (see Supplementary Table S1), while Wilcoxon tests were used as a post hoc analysis. Kaplan- Meier estimates were performed to demonstrate survival probability of threatened and non-threatened macropters when attacked by *P. fuscipes*, with predation risk and sex as variables, while a Cox proportional hazards model was used to check the significance of these variables (see Supplementary Table S3). The curves were drawn with package “survminer”^64^, and the differences between them were tested using a log-rank test.

## Supplementary Information

Supplementary Information.

## Data Availability

All data available from the Figshare. Dataset. https://doi.org/10.6084/m9.figshare.14844222.
