# Supplementary material for: Predator cue-induced plasticity of morphology and behavior in planthoppers facilitate the survival from predation
Source: Sci Rep. 2021 Aug 18;11:16760. doi: 10.1038/s41598-021-96005-3 (PMC8373946; doi:10.1038/s41598-021-96005-3)
Supplement: Supplementary file 1 — Supplementary Information. [file 41598_2021_96005_MOESM1_ESM.pdf]

Scientific Reports

Supplementary Information for:

Predator cue-induced plasticity of morphology and behavior in planthoppers facilitate the survival from predation

Jian Wen[1] and Takatoshi Ueno[1]

[1] Institute of Biological Control, Faculty of Agriculture, Kyushu University, Fukuoka, 819- 0395, Japan

Author for correspondence:

Jian Wen

E-mail: arcwenjian@gmail.com

Methods and Formulas

We calculated the proportion of macropters by formula1:

$$Pro.of\ female\ mac. = \left(\frac{no.of\ female\ mac.}{no.of\ female\ mac. + no.of\ female\ bra.}\right)\%$$

Where "pro. of female mac.", "no. of female mac." and "no. of female bra." is the proportion of female macropters, number of female macropters and number of brachypters respectively.

We calculated the proportion of surviving SBPHs (small brown planthoppers) with formula 2:

$$Pro.of\ female = \left(\frac{no.of\ female}{no.of\ female + no.of\ male}\right)\%$$

Where "pro. of female", "no. of female" and "no. of male" is the proportion of females, number of females and number of males respectively. We calculated the proportion of males by changing the numerator of formula 2 with "no. of male".

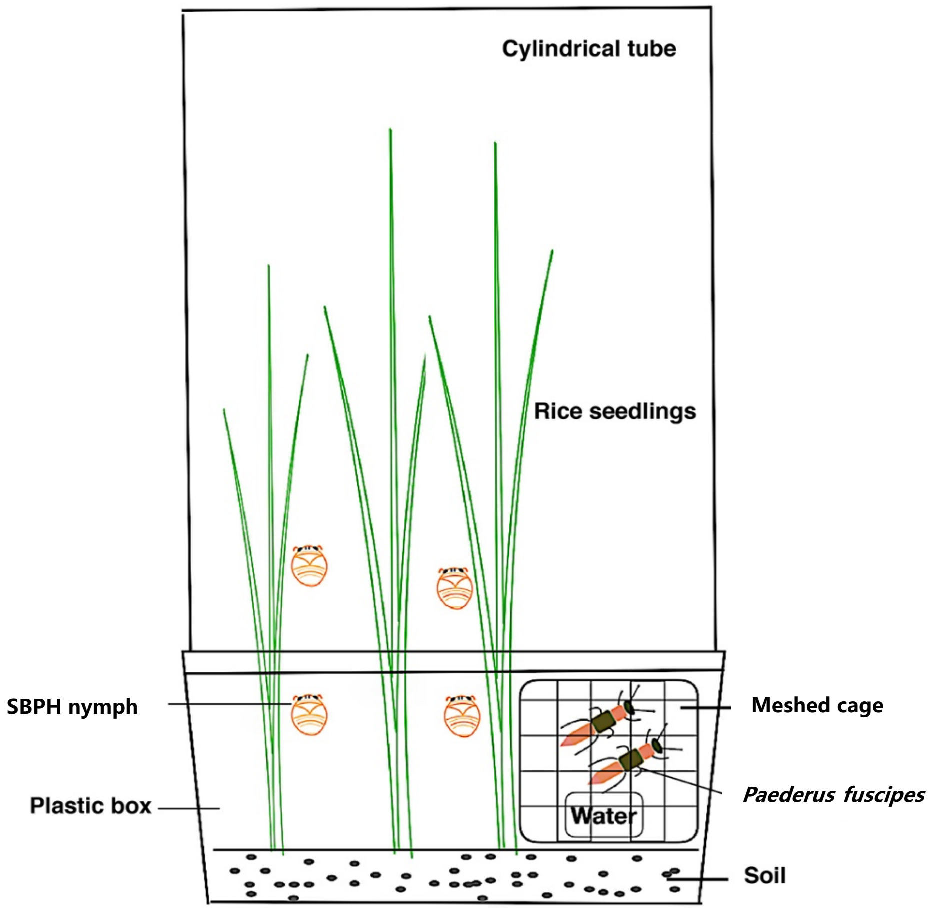

Fig. S1 A device used to produce predation risk effects of *P. fuscipes* on SBPHs. In the odor cue and visual cue treatments, the *Paederus fuscipes* in the meshed cage were replaced with cotton (soaked with predator body extract) and sealed predator cadavers, respectively.

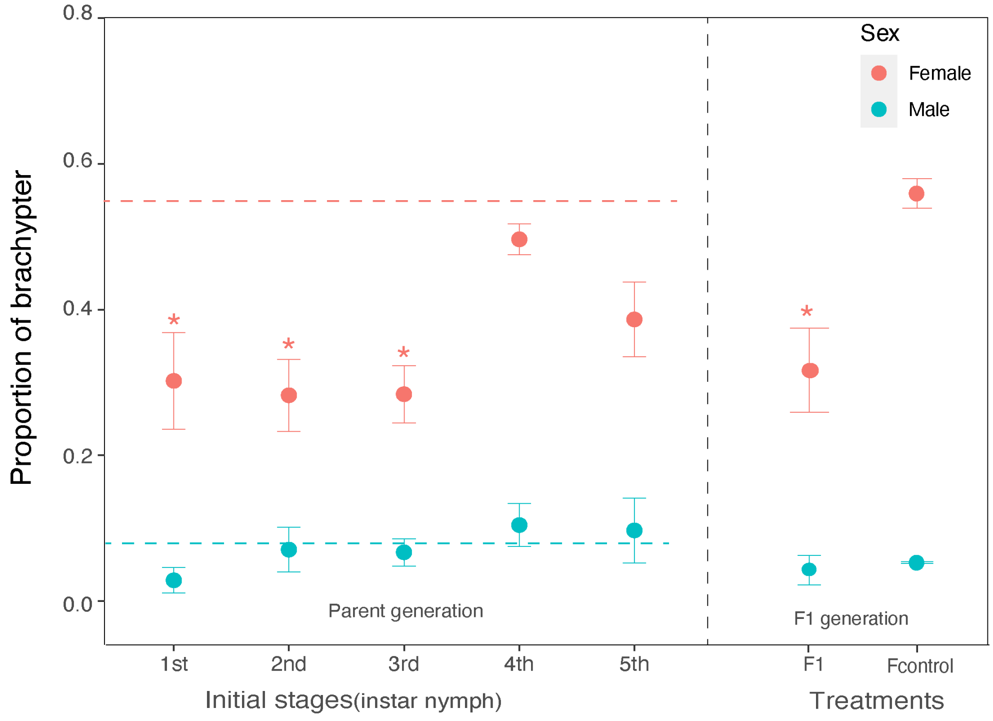

Fig. S2 Effects of predation risk (caged *P. fuscipes*) on the proportion (mean ± SE) of brachypters in parents and the F1 generation. The production of brachypters was lower than control (without caged predator). The light red and turquoise dotted lines indicate the mean proportion of female macropters and the mean proportion of male macropters in the parent control (Pcontrol) respectively. Initial stages indicate the instar stage when the nymph was exposed to predation risk. Fcontrol is the control for the F1 generation. The single asterisk above the error bar in the same color denotes a significant difference from the control (Dunnett's test, $P < 0.05$)

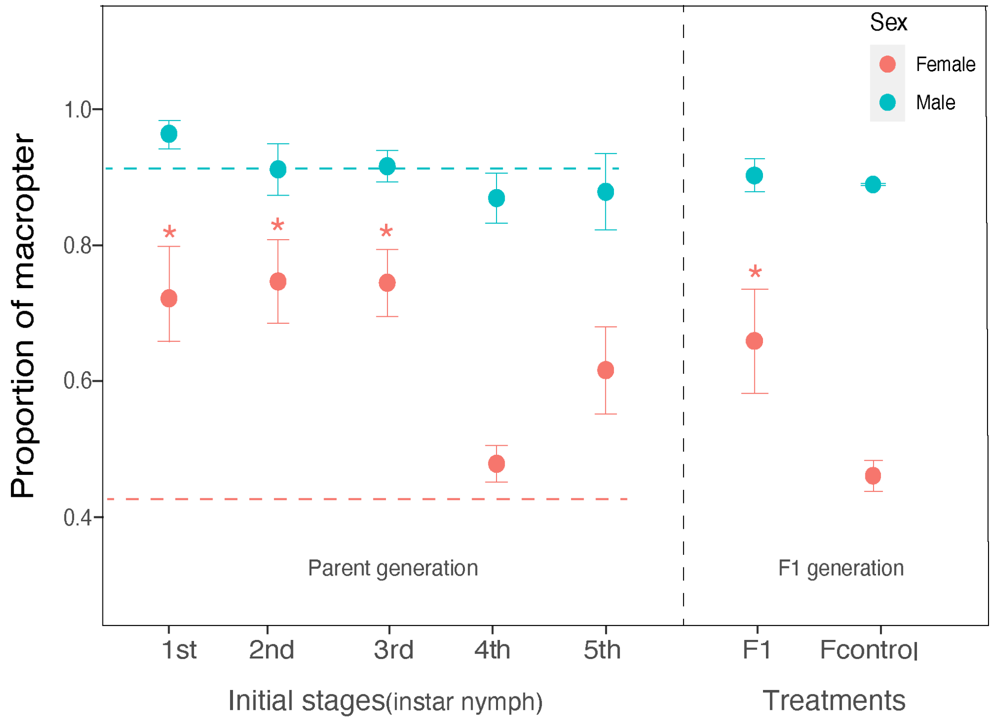

Fig. S3 Effects of predation risk (caged *P. fuscipes*) on the proportion (mean ± SE) of macropters in parents and the F1 generation. The proportion of macropters were similar when 1st, 2nd and 3rd instar nymphs were exposed to predation risk. The light red and turquoise dotted lines indicate the mean proportion of female macropters and the mean proportion of male macropters in the parent control (Pcontrol) respectively. Initial stages indicate the instar stage when the nymph was exposed to predation risk. Fcontrol is the control for the F1 generation. The single asterisk above the error bar in the same color denotes a significant difference from the control (Dunnett's test, $P < 0.05$)

Table S1 Model selection (conducted with general linear model, GLM) to explain the number of attacks by *P. fuscipes* to capture a macropter. M1- M5 were the models to analyze the number of attacks by *P. fuscipes* to capture a macropter from parent generation, while model L1- L5 were to analyze the number of attacks by *P. fuscipes* to capture a macropter from the F1 generation. No. attacks is the number of attacks by *P. fuscipes* to capture a macropter. Risk treatments included threatened macropter and non-threatened macropter (control). Sex included female and male macropter. The number of variances (K) and the AIC were provided for each model, and the model with "*" was selected based on the minimum AIC.

model	Statistical formulation	K	AIC
M1	No. attacks~ risk treatments	4	1241.1
M2	No. attacks~ sex	4	1280.6
M3	No. attacks~ risk treatments + sex	5	1231.1
M4 *	No. attacks~ risk treatments + sex+ risk treatments: sex	6	**1230.5**
L1 *	No. attacks~ risk treatments	4	**1494.5**
L2	No. attacks~ sex	4	1646.4
L3	No. attacks~ risk treatments + sex	5	1496.4
L4	No. attacks~ risk treatments + sex+ risk treatments: sex	6	1497.4

Table S2 Results of the GLM for analyzing the number of attacks by *P. fuscipes* to capture a macropter. The models were selected from Table S1 based on the minimum AIC.

Selected model	Variances	Estimate (SE)	Z value	p-value
M5: Parent generation	Intercept	2.135(0.148)	14.45	**< 0.001**
	Risk treatments	0.129 (0.148)	0.668	0.504
	Sex	0.088 (0.194)	0.937	0.349
	Risk treatments: sex	0.194 (0.121)	1.605	0.108
L1: F1 generation	Intercept	2.134 (0.050)	42.52	**< 0.001**
	Risk treatments	0.730 (0.061)	11.95	**< 0.001**

Table S3 Results of the Cox proportional hazards model for analyzing the effects of predation risk (risk treatments), sex (female and male) on the survival probability of macropter when attacked by *P. fuscipes*. Risk treatments included threatened macropter and non-threatened macropter (control). Sex included female and male macropter. The models we used for both parent and F1 generation were: time taken ~ risk treatments+ Sex.

Model	Variances	β	HR	Se (β)	0.95%CI	Z value	P-value
Parent generation	Risk treatments	-0.604	0.547	0.219	0.356- 0.839	-2.761	**0.006**
	Sex	-0.197	0.821	0.210	0.544- 1.239	-0.939	0.348
F1 generation	Risk treatments	-0.667	0.513	0.219	0.335- 0.788	-3.049	**0.002**
	Sex	-0.076	0.927	0.209	0.615- 1.40	-0.365	0.715
